# Das radiologische Gutachten – a beginner’s guide

**DOI:** 10.1007/s00117-022-00975-y

**Published:** 2022-04-20

**Authors:** Benjamin Sigl, Till Meickmann, Christian Herold

**Affiliations:** 1 Universitätsklinik für Radiologie und Nuklearmedizin, Medizinische Universität Wien, 1090 Wien, Österreich; 2 Juristische Fakultät, Universität Passau, 94032 Passau, Deutschland

## Darum geht es:

✓ Wer kann radiologische Gutachten verfassen?

✓ Was erwarten Auftraggeber (nicht) von einem Sachverständigen?

✓ Guidelines und Tipps zur Erstellung von Gutachten.

## Warum sind Gutachten wichtig und wo kann man ihnen begegnen?

Oft sind sie als unangenehme Schreibtischarbeit, anstrengende Zeitfresser und unkollegiales Fehlersuchen verpönt: radiologische Gutachten. Nichtsdestotrotz steigt die Nachfrage nach medizinischen Gutachten in den letzten Jahren ebenso wie die qualitativen Anforderungen zunehmen. Vermehrte Dokumentation, Fortschritt in der bildgebenden Diagnostik und nicht zuletzt auch ein Wandel des Arzt-Images in der Gesellschaft sind mögliche Erklärungsansätze. Entscheidungsträgern in Justiz, Versicherungen, Berufsgenossenschaften und Behörden fehlt regelmäßig die notwendige medizinische Sachkunde für Entscheidungen, die dann jedoch enorme Auswirkungen haben: für den Einzelnen, für die Gesellschaft und auch für das Gesundheitswesen. Trotz der zunehmenden Bedeutung von radiologischen Gutachten ist ihr Stellenwert in Studium und Facharztweiterbildung oft nur gering. Während in einigen Ausbildungsstandorten zumindest einzelne Gutachtennachweise (natürlich fachärztlich vidiert) zur Facharztreife vorgelegt werden müssen, ist vielerorts die Beteiligung an einer Begutachtung in der Aus- und Weiterbildungszeit nicht vorgesehen.

## Wer kommt als Gutachter in Frage?

Auch wenn für die Tätigkeit als Sachverständiger grundsätzlich keine formale Berufsqualifikation erforderlich ist, sind medizinische Sachverständige in aller Regel approbierte Ärzte mit Facharztqualifikation. Generell werden eine aktive Betätigung in dem entsprechenden Feld sowie Grundkenntnisse über die entsprechende Rechtsprechung erwartet [1, 2]. Für *gerichtliche/gerichtlich vereidigte *Sachverständige gibt es in Deutschland, Österreich und der Schweiz unterschiedliche Anforderungen, die zusätzliche Qualifikationskriterien beinhalten können [1, 3, 4]. In Österreich bedarf es beispielsweise der Eintragung in die Liste der allgemein beeideten und gerichtlich zertifizierten Sachverständigen, welche in der Regel eine Approbation, ausreichend Berufserfahrung, eine Prüfung im Verfahrensrecht und weitere, je nach Bundesland teils unterschiedliche Kriterien erfordert.

Regelmäßig setzt die gutachterliche Tätigkeit ein besonderes Maß an praktischer Erfahrung und überdurchschnittliche wissenschaftliche Kenntnisse voraus. Bei jeder Gutachtenanfrage sollte ein gewissenhafter Arzt kritisch prüfen, ob er über die notwendige Erfahrung und Kompetenz verfügt, und ggf. proaktiv die Einholung von Zusatzgutachten vorschlagen. Das gilt insbesondere für Querschnittsfächer wie die Radiologie. Aus persönlichen Gründen kommt im Einzelfall als Gutachter nicht infrage, wessen sachliche oder persönliche Unabhängigkeit in Bezug auf den konkreten Fall (bspw. bei persönlicher Verbundenheit oder Kollegen im nahen Umfeld) anzweifelbar ist.

## Welche Fragen soll ein Gutachten beantworten?

Medizinische Gutachten werden beauftragt, weil dem Auftraggeber – etwa dem Gericht – die erforderliche eigene Sachkunde fehlt, um medizinische *Sach‑, Kausalitäts-* oder *Bewertungsfragen* zu beantworten [1, 5]. Daher gibt der Auftraggeber die aus seiner Sicht entscheidungserhebliche konkrete Fragestellung und ggf. die maßgeblichen Kriterien und Annahmen für die Begutachtung vor. Der Gutachter ist an diese Vorgaben gebunden. Falls aus medizinischer Sicht Zweifel an der Sinnhaftigkeit der Fragestellung, der heranzuziehenden Kriterien oder der zugrunde gelegten Annahmen bestehen, sollten diese vor der Begutachtung mit dem Auftraggeber geklärt werden. Sollten keine klaren Fragestellungen, Kriterien oder Annahmen vorgegeben sein, ist es sinnvoll, dies festzuhalten und die angewendeten Annahmen im Gutachten eindeutig zu benennen.

*Sachfragen* können im radiologischen Tätigkeitsfeld unter anderem sein: Hat der Patient eine Hirnblutung/HWS-Distorsion/Fraktur erlitten? Während medizinische Sachfragen in aller Regel klar beantwortet werden können, ist die Beantwortung von Kausalitäts- und Bewertungsfragen deutlich komplexer. Beispiele für *Kausalitätsfragen* sind: Ist die diagnostizierte Erkrankung/Verletzung auf den Unfall/die berufliche Tätigkeit/eine Misshandlung zurückzuführen? *Bewertungsfragen* beziehen sich beispielsweise darauf, ob ein gewisser Grad der Behinderung [6] oder eine Minderung der Erwerbsfähigkeit vorliegt.

Naturgemäß hängt die Beantwortung von Kausalitäts- und Bewertungsfragen in besonderem Maße von den persönlichen Erfahrungswerten des Gutachters, dem aktuellen wissenschaftlichen Erkenntnisstand und einer ganzen Reihe weiterer Faktoren ab. Häufig wird eine Kausalitätsfrage nur mit der Angabe einer gewissen Wahrscheinlichkeit zu beantworten sein. Die Anforderungen an den Grad der Wahrscheinlichkeit variieren dabei in den unterschiedlichen Rechtsgebieten (z. B. Straf‑, Zivil- oder Sozialrecht) und je nach Perspektive (z. B. Belastung oder Entlastung). Deshalb müssen die herangezogenen Erfahrungswerte, Daten und übrigen Faktoren immer möglichst präzise benannt und ihre Aussagekraft aus medizinischer Sicht erläutert werden. Gerade bei Kausalitäts- und Bewertungsfragen muss der medizinische Gutachter nicht nur die Fragestellung selbst beantworten, sondern auch die seiner Beurteilung zugrunde gelegten Parameter für einen verständigen Leser in klar nachvollziehbarer Art und Weise darlegen. Es muss also für den medizinischen Laien überprüfbar sein, wieso der Sachverständige zu seiner Schlussfolgerung gekommen ist. Vor diesem Hintergrund empfiehlt es sich nicht nur, den Stand der Wissenschaft und die angewandten Methoden darzulegen, sondern ggf. auch eigene Erfahrungswerte klar zu benennen.

## Was erwartet der Jurist (nicht) von einem Gutachten?

Weil die fehlende eigene medizinische Sachkunde des Auftraggebers und nicht dessen fehlende Rechtskunde Anlass für die radiologische Begutachtung ist, sollte vermieden werden, rechtlich besetzte Begriffe zu verwenden oder juristische Wertungen vorzunehmen. Insbesondere gehört die Beantwortung von *Beweis-* und *Rechtsfragen* nicht zur medizinischen Gutachtertätigkeit. Es verbietet sich daher jeder Diskurs über die Sinnhaftigkeit der Fragestellung oder gar einer Rechtsnorm [7]. Gegenteiliges kann nicht nur zu Verwirrung, sondern schlimmstenfalls zur Entwertung des Gutachtens führen.

### Tipps und Kniffe beim Erstellen eines Gutachtens

**Tipp 1:** Aussagen so klar wie möglich und für Laien verständlich treffen. Im Idealfall kann ein Grad der Wahrscheinlichkeit angegeben werden.

**Tipp 2:** Über (mögliche) Befangenheit, Zeitmangel oder fehlender Expertise frühzeitig informieren und ggf. das Gutachten ablehnen.

**Tipp 3:** Vorgegebene Fragestellung und grundlegende Annahmen berücksichtigen und nicht im Gutachten hinterfragen; im Zweifel vorab mit dem Auftraggeber klären.

**Tipp 4:** Klar zwischen Sach‑, Kausalitäts- und Bewertungsfragen unterscheiden.

**Tipp 5:** Rechtliche Wertungen und rechtlich besetzte Begriffe vermeiden.

**Tipp 6:** Vergütung, Haftungsfragen und Versicherungsschutz vorab klären.

**Tipp 7:** Gegebenenfalls Einwilligung zu Untersuchungen und Entbindung von der Schweigepflicht gegenüber dem Auftraggeber in Bezug auf das Gutachten einholen.

**Tipp 8:** Neuerliche oder prophylaktische medizinische Handlungen empfehlen, falls deren Notwendigkeit (erst) im Begutachtungsprozess ersichtlich wird.

**Tipp 9:** Zusatzgutachten empfehlen, falls ein Teilbereich der gutachterlichen Tätigkeit nicht ausreichend durch die eigene Expertise abgedeckt wird.

**Tipp 10:** Gutachten abschließend noch einmal auf Vollständigkeit und Widerspruchsfreiheit prüfen.

Insgesamt sollte ein gut verwertbares Gutachten die aufgeworfenen medizinischen Fragestellungen so eindeutig wie möglich beantworten, klar strukturiert und in verständlicher Sprache verfasst sein – also insbesondere ohne übermäßige Verwendung medizinischer Fachtermini auskommen. Als Hilfsmittel zur Veranschaulichung bieten sich im radiologischen Gutachten ausgewählte Bilder an, die mit Pfeilen und Erklärungen versehen werden. In manchen Fällen können 3‑D-Rekonstruktionen zum besseren Verständnis beitragen. Für die Begutachtung kann nicht nur die aktuelle, sondern auch die zum Zeitpunkt des zu untersuchenden Vorfalls bekannte Datenlage maßgeblich sein. Das gilt sowohl für die Informationen über den Sachverhalt selbst (z. B. Informationen über den Patienten, die einem behandelnden Arzt zugänglich waren) als auch für wissenschaftliche Erkenntnisse und die gängige Behandlungspraxis. Falls vorhanden, ist es sinnvoll, auf Vorgutachten und -befunde Bezug zu nehmen, auch wenn diese die eigene Beurteilung nicht ersetzen. Ein möglicher Widerspruch sollte ausdrücklich benannt und begründet werden.

## Welchen Aufbau sollte ein Gutachten haben?

Grundlegend kann zwischen *Formulargutachten *und *Gutachten in freier Form* unterschieden werden. Atteste und Befunde zählen nicht zu Gutachten im eigentlichen Sinn. Formgutachten werden häufig von privaten Unfall- und Lebensversicherungen eingesetzt. Aus Sicht des Auftraggebers sind sie oft zeit- und kosteneffizient. Allerdings kann die Begutachtung komplexer Sachverhalte in Formularform beim Auftraggeber zu fehlerhaften Schlüssen führen. Daher ist es ggf. ratsam, dem Auftraggeber frühzeitig eine Änderung in den freien Gutachtenstil zu empfehlen. Gutachten in freier Form haben keinen vorgegebenen Aufbau, allerdings haben sich die in Tab. 1 aufgeführten Grundsätze [8] bewährt, wobei Abweichungen im Einzelfall sinnvoll sein können.

**Table 1:** 

A	Rubrum	Angaben zu Gutachter, Auftraggeber, Patient, Aktenzeichen und Datum
B	Inhaltsverzeichnis	Erleichtert eine schnelle Orientierung; insbesondere bei längeren Gutachten empfehlenswert
C	Gutachtenauftrag	Vorgegebene Fragestellung, Kriterien und Annahmen wiederholen (Formulierungen aus dem Beweisbeschluss oder Auftragsschreiben wortgetreu mit Datum übernehmen)
D	Vorliegende Unterlagen	Vorliegende Unterlagen benennen, aufgegliedert in: 1. mit dem Gutachtenauftrag übersandt, 2. vom Patienten vorgelegt, 3. vom Gutachter in Abstimmung mit dem Auftraggeber selbst beschafft
E	Sachverhalt	Darstellung des Sachverhalts unter Zugrundelegung: 1. vorliegender Unterlagen, 2. eigener Untersuchung des Patienten, 3. fremder/konsiliarischer Untersuchung bei der Begutachtung
F	Medizinische Beurteilung	Nach eigener Gliederung verfasste Gesamtbewertung: 1. bei mehreren Erkrankungskomplexen nach Bedeutung für die gutachterliche Fragestellung angeordnet, 2. innerhalb einzelner Erkrankungskomplexe chronologisch sortiert Dabei stets Erläuterung der zugrundegelegten medizinischen und wissenschaftlichen Standards, ggf. ergänzt um Angaben zu eigenen Erfahrungswerten
G	Beantwortung der Fragen an den Gutachter	Jeweils wortgenaue Wiedergabe der Fragestellung vor der Antwort, evtl. auch Übernahme der Frage in die Antwortformulierung
H	Vorschlag weiterer Begutachtungen	Falls im Gutachtenauftrag erbeten oder wenn für die Schlüssigkeit des Gutachtens unabdingbar

## Wie werden Gutachten vergütet und wer haftet?

Die Vergütung eines Gutachtens erfolgt entweder pauschal oder nach Arbeitsaufwand. Gerichtliche Gutachten werden in Deutschland aktuell je nach Komplexität der Fragestellung mit einem Stundensatz zwischen 80 und 120 € (ggf. zzgl. Umsatzsteuer) vergütet, der oft als nicht leistungsgerecht, teils nicht einmal kostendeckend angesehen wird [9]. Die Vergütung bei Privatgutachten wird daher je nach Expertise in der Regel höher angesetzt. Bestehen bereits im Vorfeld Zweifel am Umfang des Arbeitsaufwandes, wenn beispielsweise der vorgegebene Kostenrahmen deutlich überschritten wird, sollte dies idealerweise vorab mit dem Auftraggeber geklärt werden.

An dieser Stelle sei darauf hingewiesen, dass eine persönliche Haftung des Gutachters für im Zusammenhang mit seiner Tätigkeit verursachte Schäden nicht ausgeschlossen ist [1, 9]. Es empfiehlt sich, vorab mit der eigenen Versicherung zu klären, ob derartige Schäden abgedeckt sind. Patienten sind in vollem Umfang aufzuklären – auch über die fehlende gutachtenbezogene Schweigepflicht gegenüber dem Auftraggeber.

## Fazit

Die gutachterliche Tätigkeit ist ein wichtiger Bestandteil unseres Rechtssystems. Radiologinnen und Radiologen kommt eine besondere Rolle bei der Interpretation, aber auch der (manchmal begrenzten) Interpretierbarkeit von Bilddatensätzen zu. Die Bandbreite radiologischer Gutachten ist enorm und reicht von der Feststellung einzelner Unfallverletzungen über Fragen der Berufsunfähigkeit oder des Grades der Behinderung bis hin zur Beurteilung von schwerwiegenden Behandlungsfehlern. Dabei können unterschiedliche Rechtsgebiete betroffen sein, die jeweils eigene Anforderungen an Gutachten stellen. Mit Blick auf Kausalitäts- und Bewertungsfragen sollte daher der Grad der Wahrscheinlichkeit für das Vorliegen eines bestimmten Zusammenhangs ebenso klar benannt werden wie die zugrundgelegten Daten und Überlegungen.

### Literaturempfehlungen

- Leitlinie „Allgemeine Grundlagen der medizinischen Begutachtung“ [1]
- Thieme: Becher S, Ludolph E (2016) Grundlagen der ärztlichen Begutachtung, 2. Auflage
- Walter de Gruyter GmbH, Berlin/Boston: Klemm HT, Wich M (2021) Ärztliche Begutachtung
- Walter de Gruyter GmbH, Berlin/Boston: Braunschweig R (2017) Radiologische Begutachtung
- Springer: FHW Heuck FHW, Frik W, Scherz HW (1999) Radiologische Fachgutachten
- Erich Schmidt Verlag: Schönberger A, Mehrtens G, Valentin H (2016) Arbeitsunfall und Berufskrankheit: Rechtliche und medizinische Grundlagen für Gutachter, Sozialverwaltung, Berater und Gerichte, 9. Auflage
